# Direct neuronal infection of SARS-CoV-2 reveals cellular and molecular pathology of chemosensory impairment of COVID-19 patients

**DOI:** 10.1080/22221751.2021.2024095

**Published:** 2022-01-27

**Authors:** Kwang-Soo Lyoo, Hyeon Myeong Kim, Bina Lee, Young Hyun Che, Seong-Jae Kim, Daesub Song, Woochang Hwang, Sun Lee, Jae-Hoon Park, Woonsung Na, Seung Pil Yun, Yong Jun Kim

**Affiliations:** a Korea Zoonosis Research Institute, Jeonbuk National University, Iksan, Korea; b Department of Biomedical Science, Graduate School, Kyung Hee University, Seoul, Korea; c Department of Pharmacology, Institute of Health Sciences, College of Medicine, Gyeongsang National University, Jinju, Korea; d Department of Ophthalmology, Institute of Health Sciences, Gyeongsang National University School of Medicine and Gyeongsang National University Hospital, Jinju, Korea; e Department of Pharmacy, Korea University, Sejong, Korea; f Biostatistical Consulting and Research Lab, Medical Research Collaborating Center, Hanyang University, Seoul, Korea; g Department of Pathology, School of Medicine, Kyung Hee University, Seoul, Korea; h Department of Veterinary Virology, College of Veterinary Medicine, Chonnam National University, Gwangju, Korea

**Keywords:** SARS-CoV-2, viral infection, infection modelling, chemosensory impairment, hESC-derived peripheral neurons

## Abstract

Patients with recent pandemic coronavirus disease 19 (COVID-19) complain of neurological abnormalities in sensory functions such as smell and taste in the early stages of infection. Determining the cellular and molecular mechanism of sensory impairment is critical to understand the pathogenesis of clinical manifestations, as well as in setting therapeutic targets for sequelae and recurrence. The absence of studies utilizing proper models of human peripheral nerve hampers an understanding of COVID-19 pathogenesis. Here, we report that severe acute respiratory syndrome coronavirus 2 (SARS-CoV-2) directly infects human peripheral sensory neurons, leading to molecular pathogenesis for chemosensory impairments. An in vitro system utilizing human embryonic stem cell (hESC)-derived peripheral neurons was used to model the cellular and molecular pathologies responsible for symptoms that most COVID-19 patients experience early in infection or may develop as sequelae. Peripheral neurons differentiated from hESCs expressed viral entry factor ACE2, and were directly infected with SARS-CoV-2 via ACE2. Human peripheral neurons infected with SARS-CoV-2 exhibited impaired molecular features of chemosensory function associated with abnormalities in sensory neurons of the olfactory or gustatory organs. Our results provide new insights into the pathogenesis of chemosensory dysfunction in patients with COVID-19.

Most patients infected with severe acute respiratory syndrome coronavirus 2 (SARS-CoV-2) complain of abnormalities in peripheral chemosensory function, including smell and taste, as major neurological symptoms at the beginning of the infection [1]. Given the nature of typical coronavirus infection, which primarily causes a respiratory syndrome, it is unusual that SARS-CoV-2 the causative agent of coronavirus disease 2019 (COVID-19) results in peripheral neurosensory symptoms. To understand a patient's symptoms and possible sequelae, investigating the pathogenesis of the infection is necessary. SARS-CoV-2 were found to trigger the pathogenesis of abnormal olfactory function in mice by infecting non-neuronal cells such as the epithelial cells except neurons [2]. However, multiple studies including results from autopsy data of COVID-19 deaths or transgenic mice reveal the presence of virus-infected peripheral neurons in human or mice olfactory systems [3]. Hence, studies using humans are required to address the controversy surrounding cellular pathogenesis of COVID-19.

ACE2 is an entry factor for SARS-CoV-2 [4]. Since *ACE2* is not expressed in human olfactory peripheral sensory neurons [2], we reanalysed the single-cell RNA sequencing results of human olfactory neurons. We found that the expression of *ACE2* in olfactory neurons was not a complete zero, but relatively low compared to that of other genes (Figure 1(a,b) and Supplementary Table 1). As human embryonic stem cell (hESC)-derived neurons are potential alternatives to human primary neurons for modelling RNA virus infection [5], we used hESC-derived peripheral neurons to validate whether neuronal cells are involved in the COVID-19 pathogenesis. During the differentiation of peripheral neurons (Figure 1(c) and Supplementary Figs 1a-b), neural crest stem cells were stimulated with fibroblast growth factor 8 (FGF8) and retinoic acid (RA) to give frontonasal developmental signals [6], and increased number of olfactory marker protein (OMP)-expressing neurons were observed with upregulated *OMP* and downregulated *BRN3A* expression levels (Figure 1(d,e) and Supplementary Fig 1c). Along with differentiation, neurons increased *ACE2* expression, while *TMPRSS2* expression remained unchanged at the fully differentiated neuronal stage (Figure 1(f,g) and Supplementary Figs. 1d–f).

**Figure 1.**
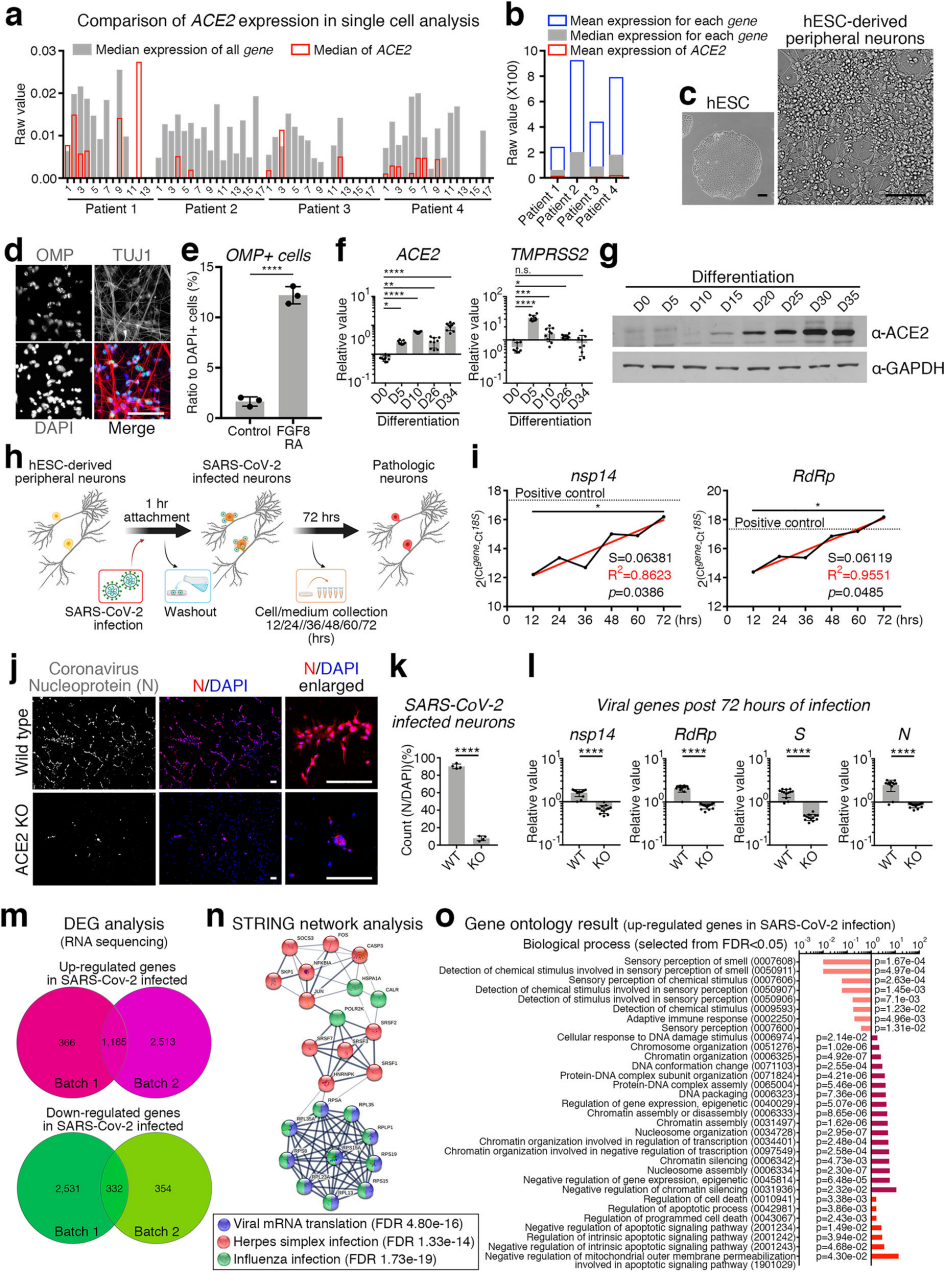
SARS-CoV-2-infected human peripheral sensory neurons reveal the cellular and molecular pathology of the chemosensory impairment. **(a)** Comparison of expression level of *ACE2* to the expression level of all other genes in single cells. All single cells were divided into 14–18 groups for each patient according to the gene expression pattern, and the median value of all cells expressing *ACE2* was indicated in red. **(b)** Comparison of expression level of *ACE2* against the mean or median of each gene. Mean values of ACE2 expression in all cells of the indicated patients are shown in red. **(c)** Representative morphology of differentiated peripheral neurons. Scale bars correspond to 100 μm. **(d)** Representative image for OMP expressing neurons. TUJ1 and DAPI was counter stained. Scale bars correspond to 50 μm. **(e)** Cell counting for OMP expressing neurons to DAPI stained nuclei in the neuronal differentiation condition with or without FGF8 and RA. Unpaired t-test, ****p < 0.0001, **(f)** Transcription level of *ACE2* and *TMPRSS2* were confirmed by qRT-PCR at each day of differentiation from hESCs. *n *= 9, biological repeat, values are mean and SD, One-way ANOVA, *p < 0.05, **p < 0.005, ***p < 0.0005, ****p < 0.0001, n.s.: non significance. **(g)** Validation of protein expression of ACE2 at each day of differentiation from hESCs. GAPDH was used as an internal control. **(h)** Schematic of SARS-CoV-2 infection strategy for hESC-derived peripheral neurons. **(i)** Tendency to increase the expression level of SARS-CoV-2 *nsp14* and *RdRp* was validated by qRT-PCR in SARS-CoV-2-infected hESC-derived peripheral neurons. Black line is measured value and red line is trend. Nonlinear regression, S = slope, R^2 ^= fitness, Pearson r, *p < 0.05. **(j)** Representative images of SARS-CoV-2 infection. Nucleoprotein of coronavirus (N) (red) was immuno-stained in wild type (upper panel) or ACE2 KO (lower panel) hESC-derived peripheral neurons. DAPI was stained as counter staining (blue). Scale bars correspond to 50 μm. **(k)** Number of SARS-CoV-2-infected neuron was counted from wild type or ACE2 KO neuros post 72 h of SARS-CoV-2 infection. *n *= 4, biological repeat, values are mean and SD. Unpaired t-test, ****p < 0.0001. **(l)** Expression level of viral genes, *nsp14*, *RdRp*, *S* and *N* were validated by qRT-PCR. *n *= 11, biological repeat, values are mean and SD. Paired t-test, ****p < 0.0001. **(m)** Venn diagram of the up-regulated genes (upper) or down-regulated genes (lower) in SARS-CoV-2-infected peripheral neurons of each group. Lists of 1,185 genes for up-regulated genes or 332 genes were shared in both experimental sets. **(n)** Network analysis using the STRING algorithm by uploading selected genes associating viral infection. Colours, categories and false discovery rate (FDR) are indicated. **(o)** Gene ontology results for biological process from up-regulated genes in SARS-CoV-2-infected neurons. Ontology terms were selected by *p*-value (FDR < 0.05). Analysed using PANTHER algorithm.

Next, we investigated whether ACE2-expressing neurons are directly targeted by SARS-CoV-2. ACE2 expressing neurons were incubated for 72 h after a one-hour-infection with SARS-CoV-2 (Figure 1(h)), and the intracellular expression levels of the viral genes *nsp14* (SARS-CoV-2 non-structural protein 14), *RdRp* (RNA-dependent RNA polymerase), *S* (spike protein) and *N* (nucleocapsid phosphoprotein) were substantially increased in the infected neurons (Supplementary Fig. 2a). Remarkably, the viral genes involved in virion replication, *nsp14* and *RdRp*, were continuously amplified within infected neurons over time, suggesting that SARS-CoV-2 propagated in the infected neurons (Figure 1(i)). In contrast to viral gene enrichment in human neurons with a viral infection, the olfactory nerves of beagle dogs were found to be protected from SARS-CoV-2 (Supplementary Fig. 2b), which is consistent with the results of a previous study indicating that SARS-CoV-2 exhibits infection specificity in humans [7]. To confirm whether SARS-CoV-2 directly infects human neurons in an ACE2-dependent manner, we generated a homozygous ACE2 *in del* knockout hESC line using CRISPR/Cas9 gene-editing technology (Supplementary Figs. 3a-f). ACE2-deficient hESCs maintained the ability to differentiate into peripheral neurons through neural crest stem cells (Supplementary Figs. 3g-i), and ACE2 not expressed as non-sense-mediated decay of mRNA occurred (Supplementary Figs. 3j-k). Subsequently, we infected the wild-type and ACE2-knockout neurons with SARS-CoV-2. The nucleoprotein of coronavirus (N) was detected in wild-type neurons but not in ACE2-knockout neurons (Figure 1(j,k) and Supplementary Figs. 3l-m), indicating that neuronal infection of SARS-CoV-2 is eliminated by blocking the ACE2-dependent direct infection pathway. Viral genes were enriched in wild-type neurons and did not increase in knockout neurons 72 h post-infection (Figure 1(l)), thus supporting the important role of ACE2 in the direct infectivity of SARS-CoV-2 into human peripheral sensory neurons.

To investigate the underlying molecular pathology due to direct neuronal infection with SARS-CoV-2, we analysed RNA sequencing results to identify genes whose expression levels were changed after SARS-CoV-2 infection in hESC-derived peripheral neurons. We established a shared list of 1,185 upregulated and 332 down-regulated genes in SARS-CoV-2-infected neurons (Figure 1(m) and Supplementary Tables 2–3) by comparing differentially expressed genes from individual experimental batches (Supplementary Fig. 4a). Interestingly, network analysis using the STRING algorithm revealed that viral infection-related genes, expression of which was up-regulated in SARS-CoV-2-infected human peripheral neurons, were thoroughly covered by categories of genes related to viral mRNA translation, herpes simplex infection, and influenza infection (Figure 1(n)). Additionally, enrichment of genes involved in the response of cells to viral infections, such as virus-induced DNA replication stress (*HSPA1A*, *HSPA1B*, and *JUN*) [8] (Supplementary Fig. 4b), virion replication (*HNRNPK* and *FKBP8*) [8] (Supplementary Fig. 4c), and mechanisms of inter-tissue infection spread (*SOCS3* and *CXCR4*) [9] (Supplementary Fig. 4d), were confirmed using quantitative PCR. In particular, the expression of the ribosomal subunit assembly-encoding gene *RPLP1*, rather than the rRNA subunits such as *RPL13* and *RPS15*, was increased following SARS-CoV-2 infection (Supplementary Fig. 4e), suggesting that the virus replication mechanism efficiently assembles the ribosome machinery during the host ribosome hijacking. We further analysed the list of shared genes using Kyoto encyclopedia of genes and genomes (KEGG). The upregulated expression of genes in SARS-CoV-2-infected neurons indicated the activation of multiple signalling pathways associated with pathogenic infections, such as viral carcinogenesis, legionellosis, herpes simplex infection, pathogenic E. coli infection, and human T-lymphotropic virus I (HTLV-I) infection (Supplementary Fig. 4g). Moreover, WNT, Rho and MAPK, which have been identified as cooperative signalling pathways for infection and replication processes of various viruses [10], were validated to be recruited into the life cycle of SARS-CoV-2 in human peripheral neurons (Supplementary Fig. 4h).

Considering that human peripheral sensory neurons express ACE2 [11] and are infected by other RNA viruses, including coronavirus [12], it is necessary to confirm whether peripheral sensory neurons develop only chemosensory dysfunction-relevant molecular pathologies rather than somatosensory dysfunction, despite infection through the entire neuronal population. To address the molecular pathology representing chemosensory abnormalities associated with the anosmia, we analysed the gene ontology using two different databases (DAVID and PANTHER). The analysis results indicated that biological processes associated with sensory impairment, such as sensory perception of smell (GO0007608), detection of chemical stimuli involved in sensory perception of smell (GO0050911), sensory perception of chemical stimuli (GO0007606), detection of chemical stimuli involved in sensory perception (GO0050907), detection of stimuli involved in sensory perception (GO0050906), detection of chemical stimuli (GO0009593), and sensory perception (GO:0007600), were negatively affected in peripheral neurons following SARS-CoV-2 infection (Figure 1(o)). Further analysis of molecular functions confirmed that SARS-CoV-2 infection attenuated the molecular mechanisms involved in peripheral sensory perception, including olfactory receptor activity (GO0004984), passive transmembrane transporter activity (GO0022803), channel activity (GO0015267), ion channel activity (GO0005216), G protein-coupled receptor activity (GO0004930), and transmembrane signalling receptor activity (GO0004888) (Supplementary Fig. 5a). To support this result, we profiled the expression of genes associated with the indicated categories in an ontology analysis. The olfactory neuronal marker genes fibronectin leucin-rich transmembrane protein (*FLRT3*), Drebrin 1 (*DBN1*), Taste 2 receptor member 31 (*TAS2R31*) (Supplementary Fig. 5b), chemosensory regulatory factors Yin Yang 1 (*YY1*), Proviral integration site for Moloney murine leukaemia virus-1 (*PIM1*), cannabinoid receptor 1 (*CNR1*) (Supplementary Fig. 5c), and axon inducing factors Cadherin EGF LAG seven-pass G-type receptor 2 (CELSR2), Cadherin EGF LAG seven-pass G-type receptor 3 (*CELSR3*), Beta-secretase 1 (*BACE1*) (Supplementary Fig. 5d) were significantly decreased in SARS-CoV-2 infected neurons. Intriguingly, expression of the chemosensory modulators *BRD2* and *EGR1*, which have been proposed as targets of feasible COVID-19 therapeutic agents, was also elevated in human peripheral neurons following SARS-CoV-2 infection (Supplementary Fig. 5e) [13,14].

Despite the undoubted value of research using human tissues to understand the pathogenesis of the disease, accessibility to patients has been extremely limited in situations such as the recent COVID-19 pandemic. To understand the pathogenesis of neurological symptoms, recent studies have reported the cellular mechanisms of nerve tissue damage upon the SARS-CoV-2 infection using transgenic mice artificially overexpressing human angiotensin-converting enzyme receptor type-2 (hACE2) to overcome the difference between species on viral infectivity [2,15]. Although the previous research has suggested that SARS-CoV-2 is inaccessible to sensory neurons and that viral infection of non-neuronal cells, excluding neurons, triggers the pathogenesis of abnormal olfactory function in mice [2,16], autopsy data from COVID-19 deaths revealed the presence of virus-infected peripheral neurons in human olfactory bulbs [3]. There lacks a consensus whether the functional abnormalities of the peripheral sensory nerves are due to infection of neuronal cells or loss of epithelium and surrounding tissues. Notably, the peripheral sensory impairment in chemosensory organs often leaves long-term sequelae in patients with COVID-19 [17], which supports the result of neuronal infection leading to a neuronal degeneration. However, the cellular and molecular pathogenesis of these clinical manifestations are still not understood. Hence, human studies are required because knowledge of inter-species differences in viral infectivity, transmission, symptoms, and pathogenesis is still insufficient. Peripheral neurons derived from human embryonic stem cells (hESCs) can be used as an alternative for research on viral infectious diseases that are constrained by the limitation of using human neural tissues [5,18]. Therefore, we used hESC-derived neurons to identify cell types that are directly involved in patient symptoms and pathological mechanisms, along with validating ACE2 expression in human olfactory neurons.

Based on the validation of the absolute expression levels of mRNA (Figure 1(a,b)) and protein (Figure 1(g) and Supplementary Fig. 4d-f), rather than relative comparisons between cell populations, we propose that peripheral neurons, including olfactory receptor neurons, express ACE2. Although 7.8% of neurons were infected with SARS-CoV-2 in the absence of ACE2, suggesting the existence of an ACE2-independent viral entry mechanism [19], most hESC-derived olfactory neurons were directly infected with SARS-CoV-2 via ACE2 (Figure 1(j,k)). These results were supported by the protrusion of molecular pathology related to olfactory dysfunction by SARS-CoV-2 infection even in mixed culture conditions with somatosensory neurons (Figure 1(o) and Supplementary Fig. 5a-e). SARS-CoV-2-infected neurons revealed ontology results, indicating a decrease in various chemosensory perception abilities. In addition, decreased transcription of developmental and function-related marker genes of olfactory nerves (Supplementary Fig. 5b), increased functional transcription factors involved in the distortion of signal perception in neurons (Supplementary Fig. 5c), and decreased neurogenesis genes required to repair damaged olfactory nerves were further confirmed (Supplementary Fig. 5d). Our results suggest that the transcriptional regulation of various genes in neurons following SARS-CoV-2 infection disrupts the balance of nerve damage and regeneration processes as well as chemosensory function, thereby disturbing the homeostasis of olfactory function.

Given the characteristics of SARS-CoV-2 which is transmitted through the respiratory tract and the localisation of the olfactory system, olfactory neurons are most likely the cells that initially infect patient body. Interestingly, we found that the culture of Vero E6 cells with the medium cultured with SARS-CoV-2-infected neurons showed cytopathic effects due to re-infection of Vero cells (TCID50 = 10^1.15^, data not shown). This means that applying a therapeutic agent to the olfactory nerve cells at an early stage of infection not only inhibits the progression of neurological symptoms, but also prevents the spread of further infections through the respiratory tract. In our study, neurons infected with SARS-CoV-2 showed increased expression of *BRD2* and *EGR1*, which are known chemosensory modulators. Interestingly, a recent study reported that, in addition to increasing the transcription of *ACE2*, BRD2 activates the transcription of viral genes by binding to the transmembrane E protein of the virus using the bromodomain. Furthermore, it has been reported that EGR1 induces an immune response and fibrosis by activating TGF beta signalling in patients with coronavirus infection. More interestingly, recent efforts to develop therapeutic agents for COVID-19 patients have proposed targeting factors, such as BRD2 and EGR1, which are increased upon SARS-CoV-2 infection. Thus, the drug under development should be applied to the olfactory system to inhibit the progression of infection.

In this study, human olfactory neurons were identified as direct infection targets of SARS-CoV-2. The use of well-manipulated human neurons is entirely advantageous for elucidation of simple pathological mechanisms. However, there is no doubt that human pluripotent stem cell-derived neurons are deficient in maturity and functionality compared which primary neurons matured by interactions with adjacent cells in animal tissues. Moreover, in vitro model systems have important limitations in measuring the functional activity of neurons owing to the lack of innervation targets. Therefore, studies using single populations should be further validated in research models with complex interactions between Schwann, epithelial, and immune cells present in the human nasal cavity. In this regard, the molecular pathology proposed in this study should be further validated in human tissues or animal models to elucidate its associations with functional abnormalities.

In summary, we report that SARS-CoV-2 directly infects human peripheral sensory neurons through the entry factor ACE2. Infected SARS-CoV-2 recruits the molecular mechanisms which are involved in the life cycle of the general virus infection. Upon viral infection of unbiased neuronal cell types, the expression of genes associated with chemosensory functions, rather than other neuronal functions, was significantly changed. These results suggest that chemosensory impairment in the olfactory or gustatory system could be induced by neuronal damage in the peripheral sensory organs of patients with COVID-19 and that loss of neuronal function should be directly targeted for treatment.

## Supplementary Material

Supplemental Material

## Materials & correspondence

ACE2 knockout hESC line (based on WA09 hESC) in this study is available from Dr. Yong Jun Kim with a completed Materials Transfer Agreement under the agreement of WiCell.

## References

[1] Mao L, Jin H, Wang M, et al. Neurologic manifestations of hospitalized patients with coronavirus disease 2019 in Wuhan, China. JAMA Neurol. 2020 Jun 1;77(6):683–690. 32275288 10.1001/jamaneurol.2020.1127 PMC7149362

[2] Brann DH, Tsukahara T, Weinreb C, et al. Non-neuronal expression of SARS-CoV-2 entry genes in the olfactory system suggests mechanisms underlying COVID-19-associated anosmia. Sci Adv. 2020 Jul 31;6(31):eabc5801. 32937591 10.1126/sciadv.abc5801 PMC10715684

[3] Meinhardt J, Radke J, Dittmayer C, et al. Olfactory transmucosal SARS-CoV-2 invasion as a port of central nervous system entry in individuals with COVID-19. Nat Neurosci. 2021 Feb;24(2):168–175. 33257876 10.1038/s41593-020-00758-5

[4] Hoffmann M, Kleine-Weber H, Schroeder S, et al. SARS-CoV-2 cell entry depends on ACE2 and TMPRSS2 and is blocked by a clinically proven protease inhibitor. Cell. 2020 Apr 16;181(2):271–280.e8. 32142651 10.1016/j.cell.2020.02.052 PMC7102627

[5] Kurapati S, Sadaoka T, Rajbhandari L, et al. Role of the JNK pathway in varicella-zoster virus lytic infection and reactivation. J Virol. 2017 Sep 1;91(17):e00640-17. 28637759 10.1128/JVI.00640-17 PMC5553188

[6] Katoh H, Shibata S, Fukuda K, et al. The dual origin of the peripheral olfactory system: placode and neural crest. Mol Brain. 2011 Sep 23;4:34. 21943152 10.1186/1756-6606-4-34 PMC3215936

[7] Munoz-Fontela C, Dowling WE, Funnell SGP, et al. Animal models for COVID-19. Nature. 2020 Oct;586(7830):509–515. 32967005 10.1038/s41586-020-2787-6 PMC8136862

[8] Wan Q, Song D, Li H, et al. Stress proteins: the biological functions in virus infection, present and challenges for target-based antiviral drug development. Signal Transduct Target Ther. 2020 Jul 13;5(1):125. 32661235 10.1038/s41392-020-00233-4 PMC7356129

[9] Sa Ribero M, Jouvenet N, Dreux M, et al. Interplay between SARS-CoV-2 and the type I interferon response. PLoS Pathog. 2020 Jul;16(7):e1008737. 32726355 10.1371/journal.ppat.1008737 PMC7390284

[10] Wehbe Z, Hammoud S, Soudani N, et al. Molecular insights into SARS COV-2 interaction with cardiovascular disease: role of RAAS and MAPK signaling. Front Pharmacol. 2020;11:836. 32581799 10.3389/fphar.2020.00836 PMC7283382

[11] Shiers S, Ray PR, Wangzhou A, et al. ACE2 and SCARF expression in human dorsal root ganglion nociceptors: implications for SARS-CoV-2 virus neurological effects. Pain. 2020 Nov;161(11):2494–2501. 32826754 10.1097/j.pain.0000000000002051 PMC7572821

[12] Verstrepen K, Baisier L, De Cauwer H. Neurological manifestations of COVID-19, SARS and MERS. Acta Neurol Belg. 2020 Oct;120(5):1051–1060. 32562214 10.1007/s13760-020-01412-4 PMC7303437

[13] Gordon DE, Jang GM, Bouhaddou M, et al. A SARS-CoV-2-human protein-protein interaction map reveals drug targets and potential drug-repurposing. bioRxiv. 2020 Mar 22. 10.1038/s41586-020-2286-9 PMC7431030 32353859

[14] Islam AMMK, Khan MAAK. Lung transcriptome of a COVID-19 patient and systems biology predictions suggest impaired surfactant production which may be druggable by surfactant therapy. Sci Rep. 2020 Nov 10;10(1):19395. 33173052 10.1038/s41598-020-76404-8 PMC7656460

[15] Winkler ES, Bailey AL, Kafai NM, et al. SARS-CoV-2 infection of human ACE2-transgenic mice causes severe lung inflammation and impaired function. Nat Immunol. 2020 Nov;21(11):1327–1335. 32839612 10.1038/s41590-020-0778-2 PMC7578095

[16] Cooper KW, Brann DH, Farruggia MC, et al. COVID-19 and the chemical senses: supporting players take center stage. Neuron. 2020 Jul 22;107(2):219–233. 32640192 10.1016/j.neuron.2020.06.032 PMC7328585

[17] Fiani B, Covarrubias C, Desai A, et al. A contemporary review of neurological sequelae of COVID-19. Front Neurol. 2020;11:640. 32655489 10.3389/fneur.2020.00640 PMC7324652

[18] Oh Y, Zhang F, Wang Y, et al. Zika virus directly infects peripheral neurons and induces cell death. Nat Neurosci. 2017 Sep;20(9):1209–1212. 28758997 10.1038/nn.4612 PMC5575960

[19] Trbojevic-Akmacic I, Petrovic T, Lauc G. SARS-CoV-2 S glycoprotein binding to multiple host receptors enables cell entry and infection. Glycoconj J. 2021 Oct;38(5):611–623. 34542788 10.1007/s10719-021-10021-z PMC8450557

