# Supplementary material for: Direct neuronal infection of SARS-CoV-2 reveals cellular and molecular pathology of chemosensory impairment of COVID-19 patients
Source: Emerg Microbes Infect. 2022 Jan 27;11(1):406–11. doi: 10.1080/22221751.2021.2024095 (PMC8803065; doi:10.1080/22221751.2021.2024095)
Supplement: Supplemental Material [file TEMI_A_2024095_SM9752.zip › TEMI 2024095_Suppl files/Supplementary_Information_vR2.0_cleancopy.docx]

**Supplementary Information**

**Direct infection of SARS-CoV-2 induces cellular and molecular pathogenesis of chemosensory impairment in hESC-derived peripheral sensory neurons.**

Kwang-Soo Lyoo, Hyeon Myeong Kim, Bina Lee, Young Hyun Che, Seong-Jae Kim, Daesub Song, Woochang Hwang, Sun Lee, Jae-Hoon Park, Woonsung Na, Seung Pil Yun, and Yong Jun Kim

**Contents**

Materials and methods

Supplementary Figure 1. Expression of ACE2 in hESC-derived peripheral sensory neurons.

Supplementary Figure 2. Human-specific direct neuronal infectivity of SARS-CoV-2.

Supplementary Figure 3. Generation of ACE2 knockout hESCs and peripheral neurons.

Supplementary Figure 4. Molecular pathology of chemosensory impairment by direct SARS-CoV-2 infection.

Supplementary Figure 5. Molecular pathology of chemosensory impairment by direct SARS-CoV-2 infection.

References

**Materials and methods**

*Human embryonic stem cell culture*

WA01 and WA09 was purchased from WiCell (Wisconsin, USA), and 02036 hiPSCs was established using OSKM-Sendai virus (Thermo Fisher, USA) in our previous study [1]. hPSCs were cultured by everyday feeding of Essential 8 medium (Thermo Fisher, USA) on the iMatrix-511 plate coating material (Matrixome, Japan) under the 5% CO_2_ condition in humidified incubator. hPSCs were passaged once a week using Versene solution (Thermo Fisher, USA), and passages within 29 to 45 were used. All the experiment using hESCs was performed under regulation KHSIRB-20-489 allowed by institutional IRB from Kyung Hee University.

*Generation of the ACE2 knockout human embryonic stem cell line*

The single guide RNA (Fig. 3a) was introduced into hESCs (WA09, passage number 29) by using NEPA21 electroporator (Nepagene, Japan). After 72 hours of electroporation, each single hESC was incubated for two weeks until colony formation. Sanger sequencing was performed to select clones which has homozygote *in del* mutation in the start codon of the ACE2 gene. All the experiment for genetic modification of human cells were performed under regulation KHUIBC (SE)-10-082 allowed by Kyung Hee University.

*Neural crest and peripheral neuron differentiation*

Colonies of hPSCs were collected and rendered into single cells using Accutase (Sigma Aldrich), and were plated on Geltrex (Thermo Fisher, USA), an artificial basement membrane-like matrix, in accordance with previous protocols [2]. To differentiate neural crest stem cells, Essential 8 medium containing 500 nM LDN193189 and 10 µM SB431542 was treated for first three days, and 3 µM CHIR99021 was added to the medium which is gradually changed to Neurobasal medium (Thermo Fisher, USA) supplemented with B27 (Thermo Fisher, USA) and N-2 (Thermo Fisher, USA). After neural crest differentiation, peripheral neurons were further differentiated by treatment of 10 nM NGF (Peprotech), 20 nM BDNF (Peprotech), 20 nM GDNF (Peprotech), 100 µM dbcAMP (Sigma Aldrich) and 100 µM sodium L-ascorbate (Sigma Aldrich) to the culture medium for additional 2 weeks.

*Virus preparation and infection*

SARS-CoV-2 (NCCP43326, strain BetaCov/Korea/KCDC03/2020) was provided by KCDC [3]. For virus replication, Vero cells were infected at a multiplicity of infection (MOI) of 0.05, and cultured in DMEM (Thermo Fisher, USA) supplemented with 2% fetal bovine serum (Thermo Fisher, USA) and penicillin-streptomycin (Thermo Fisher, USA) at 37°C, 5% CO2. To infect hESC-derived peripheral neurons, 10^4^ TCID / ml of virus were incubated for 1 hour to allow attachment, and the infected cells were incubated for an additional 72 hours after washing out the virus remaining in the medium. For animal experiment, three beagle dogs (9-month-old), serologically negative for SARS-CoV-2, were anaesthetized by intramuscular injection of Zoletil (Virbac, 10 mg/kg) and then inoculated via intranasal (i.n.) installation with SARS-CoV-2 at a dose of 10^5.5^ TCID_50_/ml in 1-ml sterile PBS. One dog was administrated 1ml DMEM as a negative control. All beagle dogs were housed separately in single cage, and sacrificed to isolate the olfactory nerves at 7 days post-inoculation (dpi). All experiments were performed at the Animal Use Biosafety Level-3 (ABL-3) facility at the Korea Zoonosis Research Institute, which is certified by the Korea Disease Control and Prevention Agency (certification number KCDC-15-3-02). The animal experiments were approved by the Institutional Animal Care and Use Committee (CBNU 2020-060), and the experimental protocols requiring biosafety were approved by the Institutional Biosafety Committee (JBNU 2020-03-001).

*Immunofluorescence staining*

Cells were fixed with 4% paraformaldehyde, and permeabilized with 0.3% Triton X‐100 (Biosesang, Seongnam, Korea) in PBS (PBST). Subsequently, Cells were blocked with 5% normal goat serum (Jackson Laboratory, Bar Harbor, Maine, USA) in PBST. Cells were then incubated with primary antibodies overnight at 4°C followed by incubation with secondary antibodies for 1 hour at RT. All primary antibodies were diluted in PBS with 0.1% normal serum, and secondary antibodies were used at a 1:500 dilution in PBS. Slides were mounted using the Vectashield mounting medium with DAPI (Vector labs), and were imaged using the fluorescence microscopy (Olympus Corporation, Tokyo, Japan).

*RNA sequencing and informatic analysis*

Total RNAs were separately prepared from two batches of uninfected (n = 2) and SARS-CoV-2 infected (n = 2) peripheral neurons. The libraries were prepared for 150 bp paired-end sequencing using TruSeq Stranded Total RNA Sample Prep Kit with Ribo-Zero H/M/R (Illumina, CA, USA). Purified 1ug of total RNA molecules were fragmented, and single-stranded cDNAs was synthesized by random hexamer priming. Using this single-stranded cDNAs as a template for second strand, double-stranded cDNA was further generated. After sequential process of end repair, A-tailing and adapter ligation, cDNA libraries were amplified using PCR (Polymerase Chain Reaction). Quality of these cDNA libraries was evaluated with the Agilent 2100 BioAnalyzer (Agilent, CA, USA), and libraries were quantified using the KAPA library quantification kit (Kapa Biosystems, MA, USA) according to the manufacturer’s library quantification protocol. After the cluster amplification of denatured templates, sequencing was progressed as paired-end (2×150bp) using Illumina Novaseq6000 (Illumina, CA, USA). To analyse, FASTQ files were uploaded into Partek Flow server (Partek Inc.), and raw reads were quantified to hg19 (Ensembl Transcripts release 75) using the Bowtie 2 aligner. Normalized read counts using Transcripts Per Million (TPM) methods were statistically modeled using Partek Flow’s Gene Specific Analysis (GSA) approach. Then, lists of up-regulated or down-regulated genes in SARS-CoV-2 infected neurons were derived using differential analysis filter followed by comparing analysis for both lists from separate batches. Differentially expressed genes were further analysed by using DAVID and PANTHER ontology database. RNA sequencing data generated in this study have been deposited in the Gene Expression Omnibus (GEO) database, GSE161281. Re-analysis of human *ACE2* expression using single cell RNA sequencing results was performed using deposited data set of GSE139522. Re-analysis of human ACE2 expression using single cell RNA sequencing results was performed using deposited data set of GSE139522. Single cell count matrix was downloaded from (<https://www.ncbi.nlm.nih.gov/geo/query/acc.cgi?acc=GSE139522>). Genes that expressed in less than 4 cells were filtered. In addition to this, cells that show less than 200 expressed genes, more than 2,500 expressed genes or mitochondrial genes expressed ratio was over 5 percent were filtered. Read counts were Log-Normalized and cell clusters were discovered using PCA (principal component analysis) and graph based clustering algorithm. Processing of the read count matrix was performed using Seurat package. To analyse all cells expressing the minimum amount of mRNA, all genes with a non-zero expression level were selected, and the number of these genes was 21,418 in patient 1, 22,944 in patient 2, 24,381 in patient 3, and 22,941 in patient 4. The number of cells expressing these genes was 2,634 in patient 1, 10,809 in patient 2, 5,266 in patient 3, and 11,962 in patient 4. Median expression levels of all detected genes and ACE2 expression levels in all cells were compared, and clustered into groups with similar expression patterns using PCA. The number of separated cell clusters was 14 in patient 1, 17 in patient 2, 18 in patient 3, and 17 in patient 4. The average expression level of ACE2 in each cluster was compared with the median expression value of all gene expression levels of cells in the cluster.

*Quantitative real time PCR*

Total RNAs from cultured cells were extracted using RNA isolation kit (Qiagen, Valencia, CA, USA) according to the manufacturer’s protocol. RNA concentration was measured spectrophotometrically using a Denovix DS-11 spectrophotometer (Denovix Inc., USA). The cDNA was synthesized using HiSenScript™ RH (-) RT PreMix kit (iNtRON, Seongnam, South Korea) by adding 2 μg of RNA to RT premix tube followed by the reaction step with reverse transcriptase at 50 °C for 1hour and inactivation step at 85 °C for 10 minutes in the T100 thermal cycler (Bio-Rad, Hercules, CA, USA). Quantitative real-time PCR (qRT-PCR) was performed in duplicate or triplicate for each sample using iQ SYBR Green Supermix (Bio-Rad) and CFX96 Real-Time PCR machine (Bio-Rad) with gene specific primer sets (Supplementary Table 4). There were no missing data, and number of biological repeats are indicated in corresponding figure legends.

*Quantification, statistical analysis, and software*

Data was statistically analysed using Graphpad Prism 7. Means +/- standard deviation is plotted and p-values were calculated using one-way ANOVA, paired t-test and unpaired t-test as noted. The images were created using resources of BioRender.com.

**Supplementary Figure 1**

**
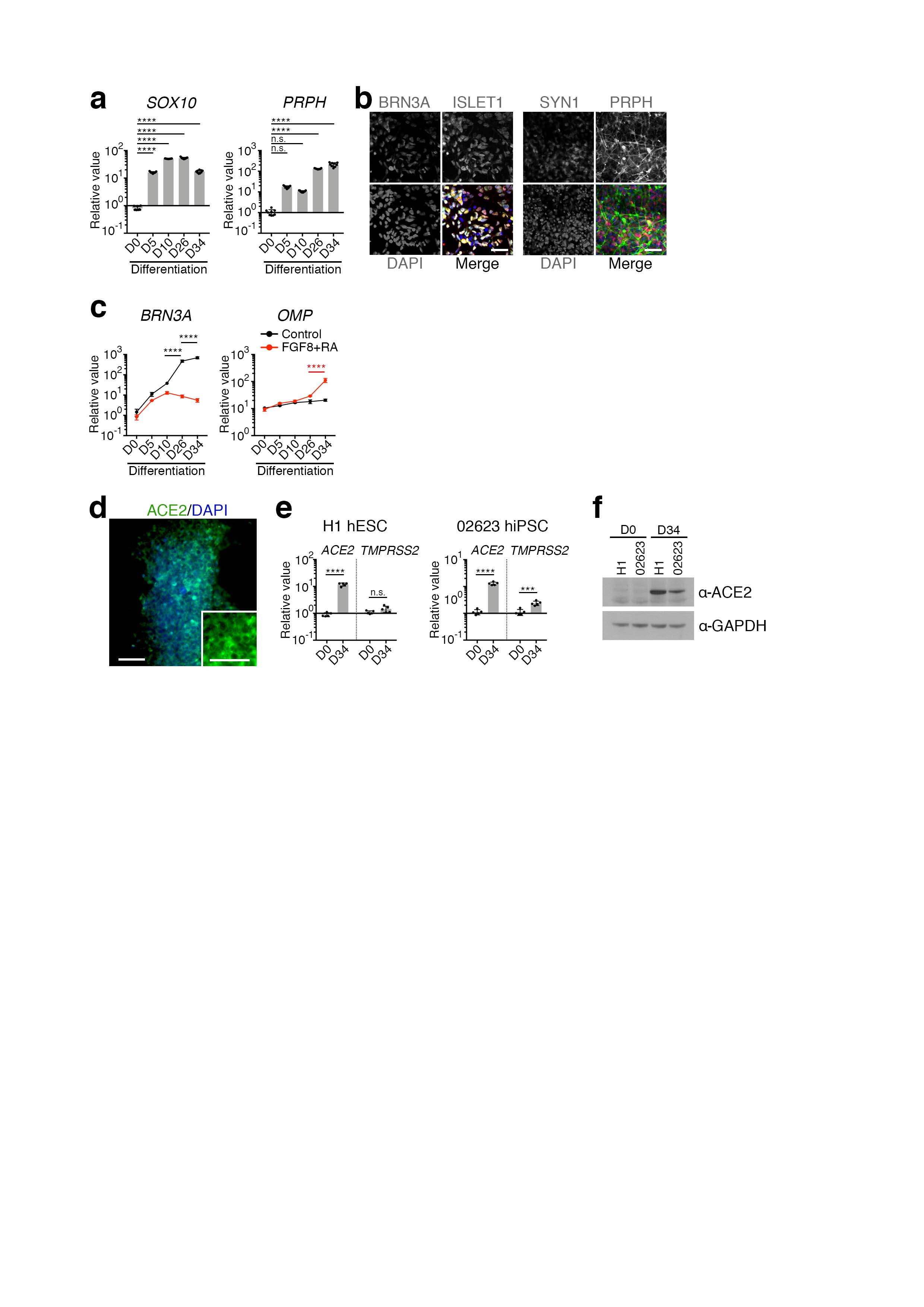
**

**Supplementary Figure 1. Expression of ACE2 in hESC-derived peripheral sensory neurons.**

**(a)** Transcription level of *SOX10* and *PRPH* were confirmed by qRT-PCR (quantitative real-time PCR) at each day of differentiation from hESCs. *n*=9, biological repeat, values are mean and SD, One-way ANOVA, *****p*<0.0001, n.s.=non significance.

**(b)** Immunofluorescence images for peripheral neuron markers, BRN3A (red), ISLET1 (green), DAPI (blue) and merge (upper panel), SYN1 (red), PRPH (green), DAPI (blue) and merge (lower panel). Scale bars correspond to 50 μm.

**(c)** Transcription level of *BRN3A* and *OMP* were traced by qRT-PCR at each day of differentiation from hESCs with or without FGF8 and RA. *n*=3, biological repeat, values are mean and SD, One-way ANOVA, *****p*<0.0001.

**(d)** Representative image for ACE2 expression (green) of differentiating cells at day 10, and an enlarged image in a bottom-right small box. Scale bars correspond to 50 μm.

**(e)** Transcription level of *ACE2* and *TMPRSS2* of H1 hESC and 02036 hiPSC lines at the day 0 or day 34 after differentiation. *n*=5, biological repeat, values are mean and SD, One-way ANOVA, ****p*<0.0005, *****p*<0.0001, n.s.=non significance.

**(f)** Validation of protein expression of ACE2 at each day of differentiation from H1 hESCs or 02623 hiPSCs. GAPDH was used as an internal control.

**Supplementary Figure 2**

**
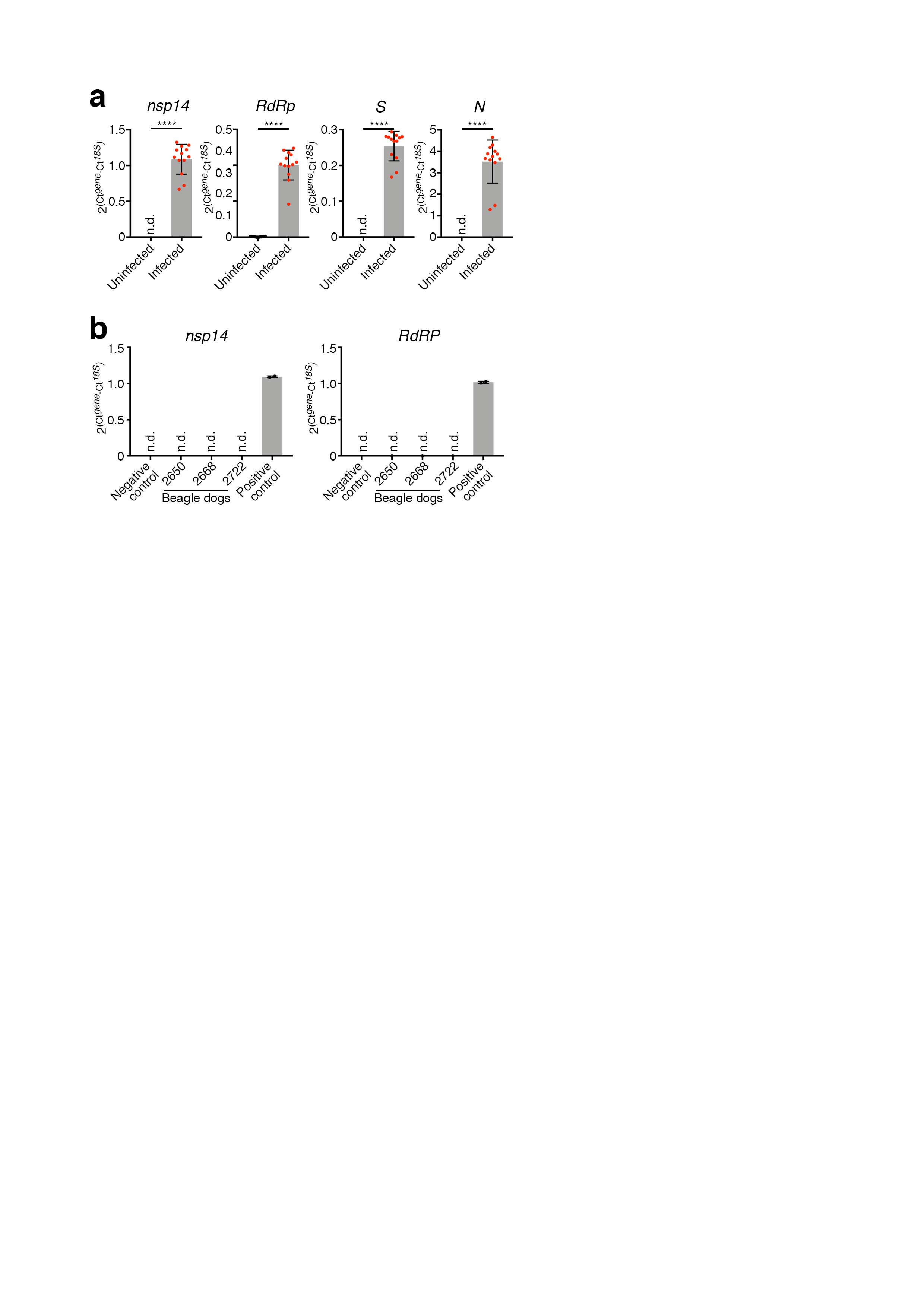
**

**Supplementary Figure 2. Human-specific direct neuronal infectivity of SARS-CoV-2.**

**(a)** Expression of viral genes, *nsp14*, *RdRp*, *S* and *N* in uninfected or SARS-CoV-2 infected hESC-derived peripheral neurons were validated after 72 hours of infection by qRT-PCR. *n*=11, biological repeat, values are mean and SD. Unpaired t-test, *****p*<0.0001, n.d.=not determined.

**(b)** Detection of SARS-CoV2 *nsp14* and *RdRp* by qRT-PCR after 72 hours of SARS-CoV-2 infection into olfactory nerve of beagle dogs. *n*=3 for each beagle dog, technical repeat, values are mean and SD., n.d.=not determined.

**Supplementary Figure 3**

**
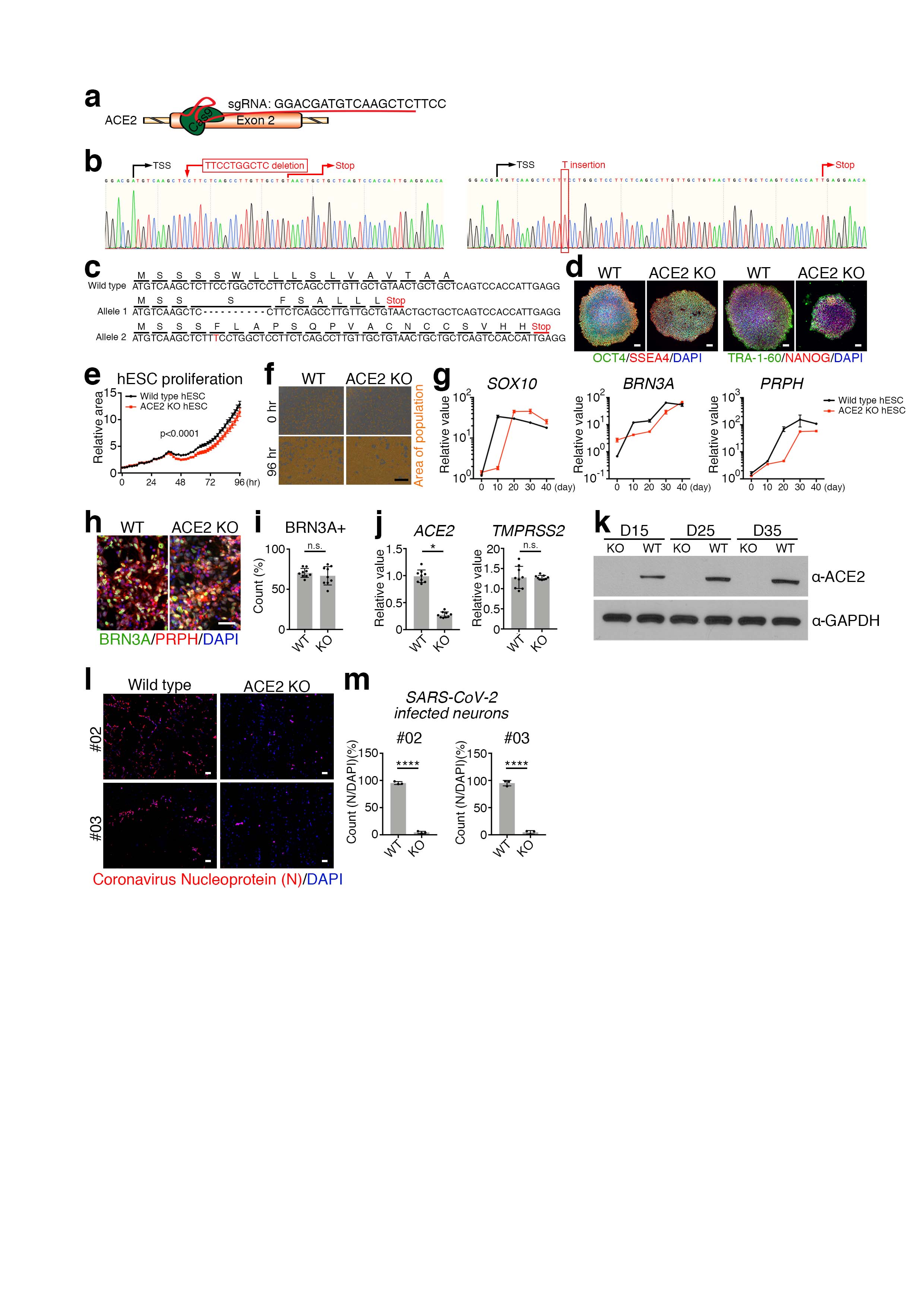
**

**Supplementary Figure 3. Generation of ACE2 knockout hESCs and peripheral neurons.**

**(a)** Schematic of *in del* knockout for ACE2 in hESC with indicating sgRNA sequence.

**(b-c)** Sanger sequencing results of ACE2-knockout hESCs. Stop codon generating non-sense mutation is indicated in each allele with sites of nucleotides deletion or single nucleotide insertion.

**(d)** Immunofluorescence images of wild type and ACE2 KO hESCs with OCT4 (green), SSEA-4 (red) and DAPI (blue)(left panel), and TRA-1-60 (green), NANOG (red) and DAPI (blue)(right panel). Scale bars correspond to 50 μm.

**(e)** Proliferation of wild type and ACE2 KO hESCs were measured by live imaging-based calculation.

**(f)** Represent images at 96 hours of proliferation. Yellow area covered cell population. Scale bars correspond to 500 μm.

**(g)** Transcription level of *SOX10*, *BRN3A* and *PRPH* were confirmed by qRT-PCR at each day of differentiation from wild type and ACE2 KO hESCs. *n*=9, biological repeat, values are mean and SD.

**(h)** Immunofluorescence images with expression of marker proteins, BRN3A (green), PRPH (red) and DAPI (blue), for differentiated peripheral neurons from wild type and ACE2 KO hESCs. Scale bars correspond to 50 μm.

**(i)** Number of BRN3A expressing peripheral neurons were counted. *n*=9, biological repeat, values are mean and SD. Unpaired t-test. n.s.=non significance.

**(j)** Transcription level of *ACE2* and *TMPRSS2* mRNA in wild type and ACE2 KO hESCs were validated by qRT-PCR. *n*=9, biological repeat, values are mean and SD. Unpaired t-test, **p*<0.05, n.s.=non significance.

**(k)** Immunoblotting to verify deficiency of ACE2 in KO hESCs at each day of differentiation. Arrow is indicating proper band for ACE2. GAPDH was used for internal control.

**(l)** Representative images for SARS-CoV-2 infection in biological repeated clones. Nucleoprotein of coronavirus (N) (red) was immuno-stained in wild type (upper panel) or ACE2 KO (lower panel) hESC-derived peripheral neurons. DAPI was stained as counter staining (blue). Scale bars correspond to 50 μm.

**(m)** Number of SARS-CoV-2 infected neuron was counted from wild type or ACE2 KO neuros post 72 hours of SARS-CoV-2 infection. *n*=3, biological repeat, values are mean and SD. Unpaired t-test, *****p*<0.0001.

**Supplementary Figure 4**

**
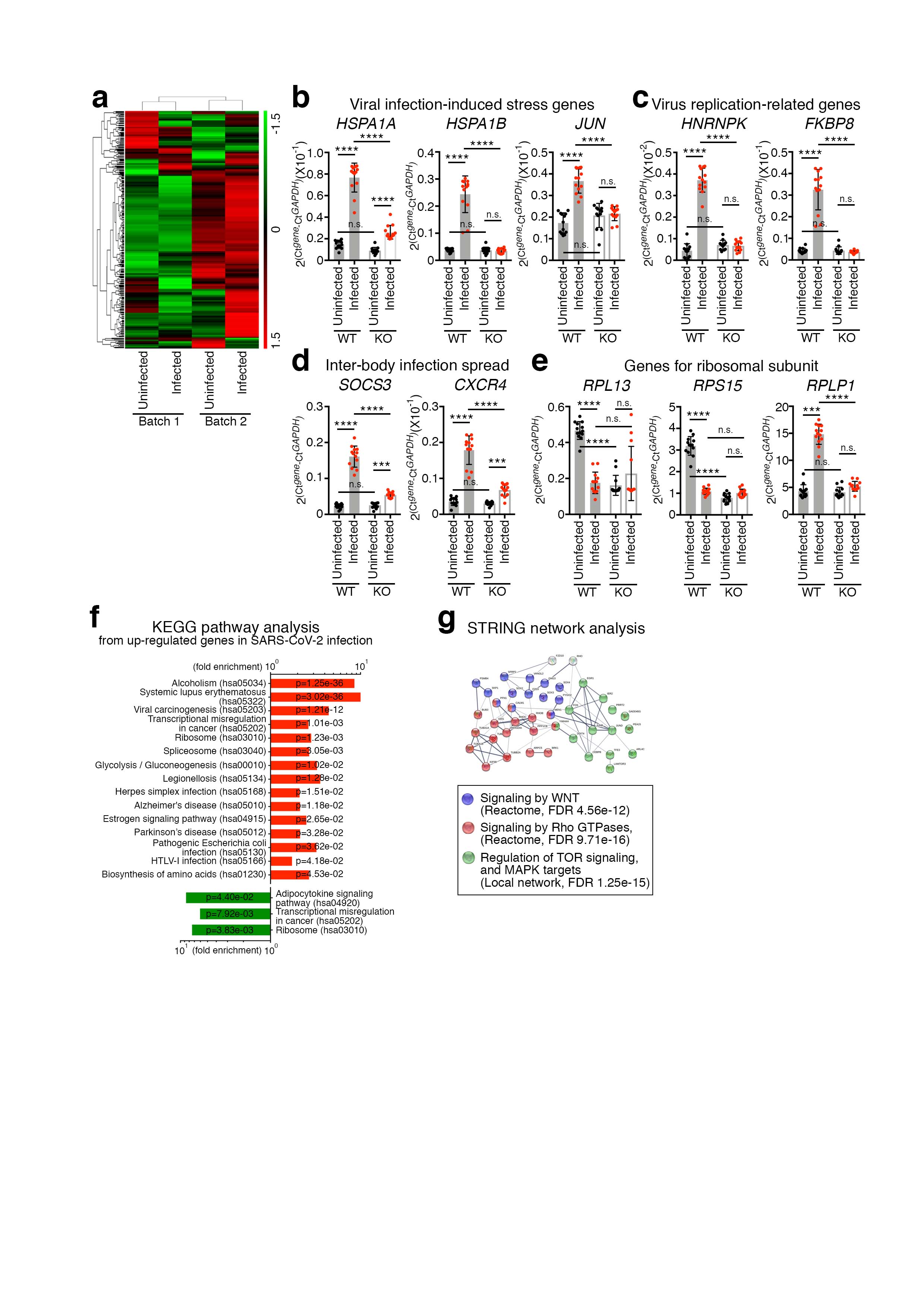
**

**Supplementary Figure 4. Molecular pathology of direct SARS-CoV-2 infection into hESC-derived peripheral sensory neurons.**

**(a)** Unbiased heat map representation of supervised hierarchical clustering of each differentiation condition.

**(b)** Validation of expression level of genes related to viral infection-induced stress by qRT-PCR. *n*=11, biological repeat, values are mean and SD. Unpaired t-test, *****p*<0.0001, n.s.=non significance.

**(c)** Validation of expression level of genes related to virus replication by qRT-PCR. *n*=11, biological repeat, values are mean and SD. Unpaired t-test, *****p*<0.0001, n.s.=non significance.

**(d)** Validation of expression level of genes related to infection spread and virus-induced cell death by qRT-PCR. *n*=11, biological repeat, values are mean and SD. Unpaired t-test, ****p*<0.0005, *****p*<0.0001, n.s.=non significance.

**(e)** Validation of expression level of genes related to ribosomal machinery by qRT-PCR. *n*=11, biological repeat, values are mean and SD. Unpaired t-test, ****p*<0.0005, *****p*<0.0001, n.s.=non significance.

**(f)** KEGG analysis result with shared up- (red bars) or down- (green bars) regulated gene list. Pathway terms were selected by p-value < 0.05.

**(g)** Network analysis using the STRING algorithm by uploading selected genes associating signal transduction[4-6]. Colors, categories and false discovery rate (FDR) are indicated.

**Supplementary Figure 5**

**
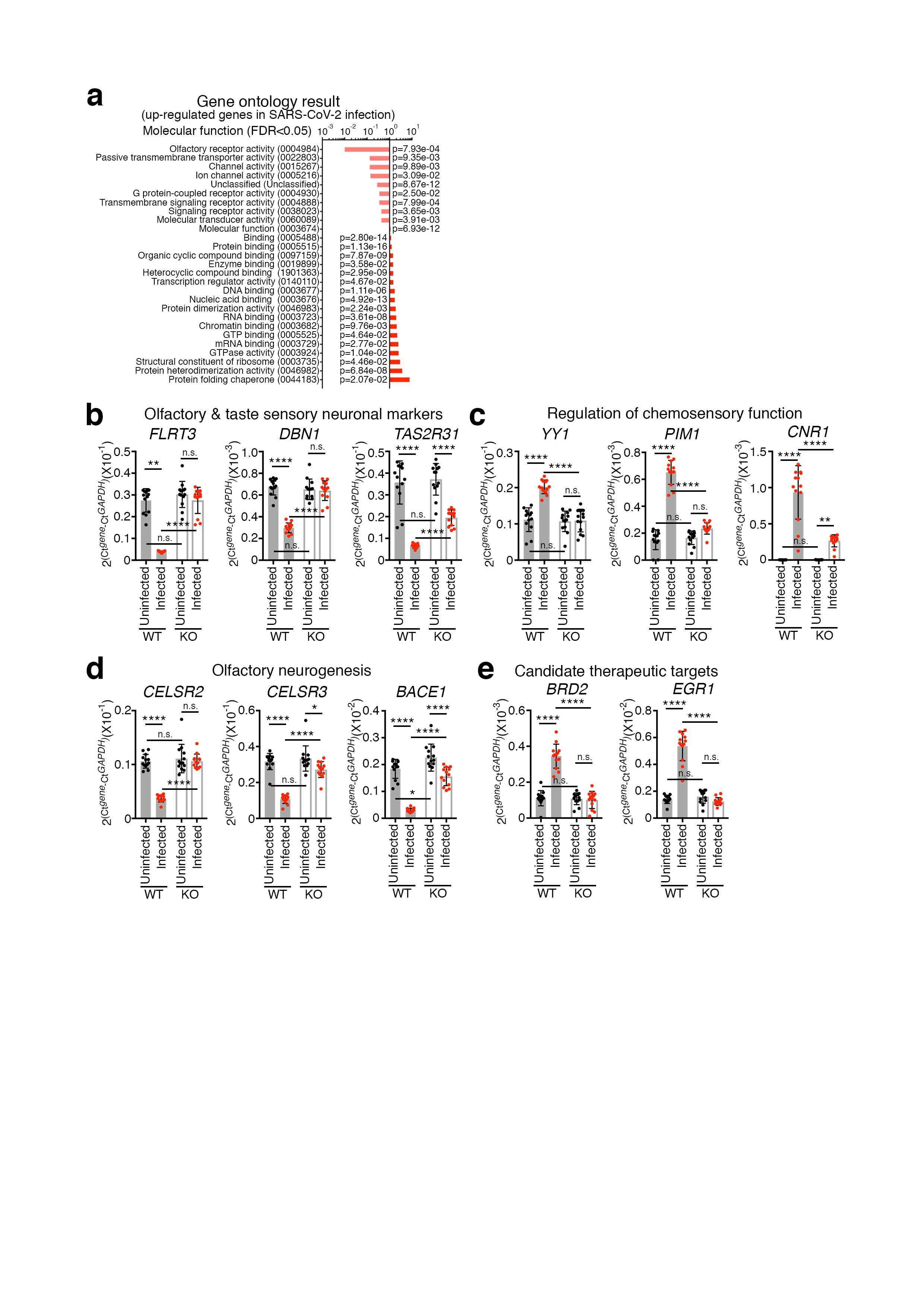
**

**Supplementary Figure 5. Molecular pathology of chemosensory impairment by direct SARS-CoV-2 infection.**

**(a)** Gene ontology results for molecular function from up-regulated genes in SARS-CoV-2 infected neurons. Ontology terms were selected by p-value (FDR<0.05). Analysed using PANTHER algorithm.

**(b)** Gene expression for olfactory or taste neuronal markers was validated using qRT-PCR. *n*=11, biological repeat, values are mean and SD. Unpaired t-test, ***p*<0.005, *****p*<0.0001, n.s.=non significance.

**(c)** Gene expression for chemosensory regulators was validated using qRT-PCR. *n*=11, biological repeat, values are mean and SD. Unpaired t-test, ***p*<0.005, *****p*<0.0001, n.s.=non significance.

**(d)** Gene expression for olfactory neurogenesis factors was validated using qRT-PCR. *n*=11, biological repeat, values are mean and SD. Unpaired t-test, **p*<0.05, *****p*<0.0001, n.s.=non significance.

**(e)** Gene expression for putative COVID-19 therapeutic targets were validated using qRT-PCR. *n*=11, biological repeat, values are mean and SD. Unpaired t-test, *****p*<0.0001, n.s.=non significance.

**References**

1 Mukherjee-Clavin B, Mi R, Kern B, Choi IY, Lim H, Oh Y, Lannon B, Kim KJ, Bell S, Hur JK, Hwang W, Che YH, Habib O, Baloh RH, Eggan K, Brandacher G, Hoke A, Studer L, Kim YJ, Lee G. Comparison of three congruent patient-specific cell types for the modelling of a human genetic Schwann-cell disorder. Nat Biomed Eng 2019; 3: 571-82

2 Kim YJ, Lim H, Li Z, Oh Y, Kovlyagina I, Choi IY, Dong X, Lee G. Generation of multipotent induced neural crest by direct reprogramming of human postnatal fibroblasts with a single transcription factor. Cell Stem Cell 2014; 15: 497-506

3 Kim JM, Chung YS, Jo HJ, Lee NJ, Kim MS, Woo SH, Park S, Kim JW, Kim HM, Han MG. Identification of Coronavirus Isolated from a Patient in Korea with COVID-19. Osong Public Health Res Perspect 2020; 11: 3-7

4 More S, Yang X, Zhu Z, Bamunuarachchi G, Guo Y, Huang C, Bailey K, Metcalf JP, Liu L. Regulation of influenza virus replication by Wnt/beta-catenin signaling. PLoS One 2018; 13: e0191010

5 Van den Broeke C, Jacob T, Favoreel HW. Rho'ing in and out of cells: viral interactions with Rho GTPase signaling. Small GTPases 2014; 5: e28318

6 Kumar R, Khandelwal N, Thachamvally R, Tripathi BN, Barua S, Kashyap SK, Maherchandani S, Kumar N. Role of MAPK/MNK1 signaling in virus replication. Virus Res 2018; 253: 48-61
